# Bayesian Estimation of Phase Dynamics Based on Partially Sampled Spikes Generated by Realistic Model Neurons

**DOI:** 10.3389/fncom.2017.00116

**Published:** 2018-01-08

**Authors:** Kento Suzuki, Toshio Aoyagi, Katsunori Kitano

**Affiliations:** ^1^Department of Complexity Science and Engineering, Graduate School of Frontier Sciences, University of Tokyo, Kashiwa, Japan; ^2^Laboratory for Neural Circuit Theory, RIKEN Brain Science Institute, Wako, Japan; ^3^Graduate School of Informatics, Kyoto University, Kyoto, Japan; ^4^Department of Human and Computer Intelligence, Ritsumeikan University, Kusatsu, Japan

**Keywords:** coupled oscillators, phase dynamics, multi-neuronal spikes, Bayesian estimation, connectivity inference

## Abstract

A dynamic system showing stable rhythmic activity can be represented by the dynamics of phase oscillators. This would provide a useful mathematical framework through which one can understand the system's dynamic properties. A recent study proposed a Bayesian approach capable of extracting the underlying phase dynamics directly from time-series data of a system showing rhythmic activity. Here we extended this method to spike data that otherwise provide only limited phase information. To determine how this method performs with spike data, we applied it to simulated spike data generated by a realistic neuronal network model. We then compared the estimated dynamics obtained based on the spike data with the dynamics theoretically derived from the model. The method successfully extracted the modeled phase dynamics, particularly the interaction function, when the amount of available data was sufficiently large. Furthermore, the method was able to infer synaptic connections based on the estimated interaction function. Thus, the method was found to be applicable to spike data and practical for understanding the dynamic properties of rhythmic neural systems.

## Introduction

**Figure 1:**
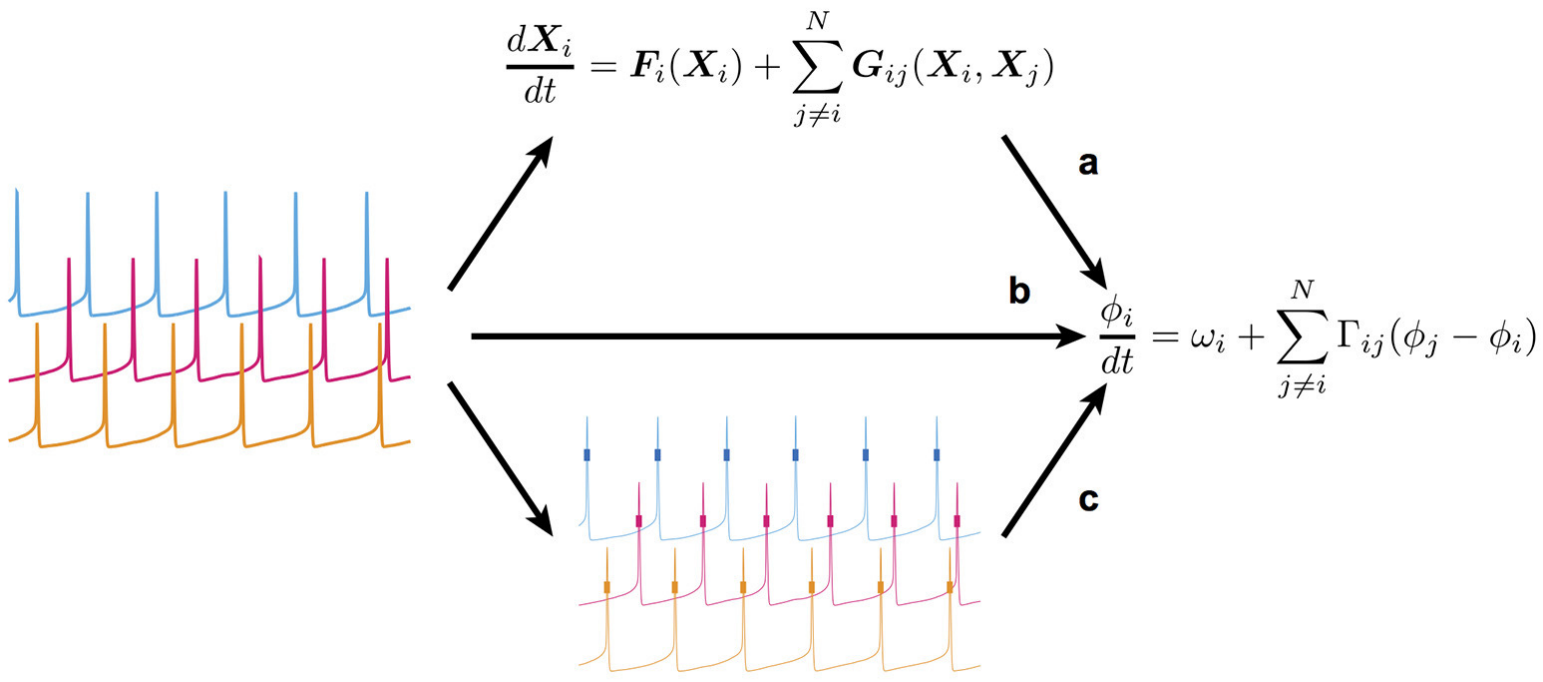
Scheme of the present study's framework. **(a)** The conventional way in which a detailed model of coupled nonlinear oscillators is first constructed, and then reduced (simplified) to retain only the essential dynamics. **(b)** The method used to extract the reduced dynamic model directly from time-series data obtained by measurement of the oscillatory system. **(c)** The method used in this study to estimate the reduced dynamic model from spike data. Note that membrane potentials (thin lines) were unavailable in this method. Therefore, only spikes (short thick lines) could be used.

Rhythmic activities in neurons and neuronal networks are thought to contribute to information transfer and processing in the brain. Specific frequency bands in rhythmic activities contribute to or disrupt neural information transfer from one neuron to another or from one brain region to another (Mallet et al., 2008; Sohal et al., 2009; Jackson et al., 2011; Liebe et al., 2012; McGinn and Valiante, 2014; Bastos et al., 2015). Synchronization, desynchronization, and phase-locking in rhythmic activities might play an important role in neural information representation and processing (Womelsdorf et al., 2006, 2007; van Elswijk et al., 2010; van Wingerden et al., 2010; Vinck et al., 2010; Park et al., 2013). To understand the mechanisms underpinning such functions, it is necessary to mathematically describe and analyze the properties of the underlying nonlinear dynamics (Hoppensteadt and Izhikevich, 1997). A mathematical description of this sort can be achieved with a detailed model based on biophysical mechanisms such as the kinetics of ion channels responsible for a given change in neuronal membrane potential (Hodgkin and Huxley, 1952). Such a model generally consists of a considerable number of variables and parameters. While such a detailed model enables us to directly compare its behavior with experimental data recorded under various experimental conditions, it is usually too complicated to extract essential properties of the dynamic system. As long as the detailed model possesses the nature of a nonlinear oscillator, however, its complicated dynamics can be reduced to those described by a single variable, the phase (Ermentrout and Kopell, 1984; Kuramoto, 1984; Hansel et al., 1995). Although this reduction makes impossible a quantitative comparison of the behavior of the reduced phase dynamics with experimentally observed phenomena, it provides a mathematically tractable framework through which to understand the essential properties of the dynamic system (see Figure 1a). Thus, most previous studies have first described the detailed dynamics of the model before reducing it to the simpler phase dynamics in order to analyze its dynamic properties as a rhythmic system.

However, it is quite difficult to exactly construct a detailed model based on experimental data due to nonlinearity, model selection, and parameter tuning. Therefore, it is best to obtain the reduced phase dynamics directly from experimental data if possible (Figure 1b; Tokuda et al., 2007; Kralemann et al., 2008; Cadieu and Koepsell, 2010; Stankovski et al., 2012). Ota and Aoyagi (2014) proposed a method by which one can extract the phase dynamics directly from the time-series data of coupled nonlinear oscillators using Bayesian estimation. In the proposed method, it is assumed that time-series data of all units composing a coupled oscillatory system are available. On applying the method to neural activity, however, simultaneous recoding of membrane potentials and time-series data of neuronal states from multi-neurons is still difficult, and only multi-neuronal spikes that represent a specific timing or phase in oscillatory activity can be obtained. Therefore, it remains to be established whether the proposed Bayesian approach is an effective means of extracting the phase dynamics from multi-neuronal spike data. In the present study, we addressed this issue by applying the Bayesian approach to the simulated spike data of a conductance-based neuronal network model (Figure 1c). Using the simulated spike data, we were able to compare the phase dynamics estimated directly from the multi-neuronal spike data with those theoretically derived from the detailed model.

## Materials and methods

We first conducted a numerical simulation of the computational neuronal network model in order to generate multi-neuronal spike data. We also theoretically derived the phase dynamics from the detailed model of the neuronal network with the phase reduction technique (see *Phase response analysis*). We then applied the Bayesian estimation to the simulated spike data and confirmed the validity of the estimated results in comparison with the reduced theoretical model (Ota and Aoyagi, 2014).

### Simulation model of a neuronal network

Our network model consisted of globus pallidus (GP) neurons in the basal ganglia, which exhibit autonomous periodic firing. We used the conductance-based model of a GP neuron previously proposed by Fujita et al. (2012) (accession number 143100 in ModelDB). The membrane potential dynamics of the *i*th neuron *V*_*i*_ is represented by the following equation:

(1) $$\begin{array}{l} C_{m}\frac{dV_{i}}{dt}=-g_{\text{leak}}\left(V_{i}-E_{\text{leak}}\right)-I_{\text{NaF}}-I_{\text{NaP}}-I_{\text{Kv2}}-I_{\text{Kv3}}\\ \;-I_{\text{Kv4f}}-I_{\text{Kv4s}}-I_{\text{KCNQ}}-I_{\text{CaH}}-I_{\text{HCN}}-I_{\text{SK}}\\ \;+I_{\text{syn},\text{i}}+I_{\text{app},\text{i}}+\eta_{i}(t), \end{array}$$

where *C*_m_, *g*_leak_, and *E*_leak_ are the unit capacitance, leak conductance, and reversal potential of the leak current, respectively. *I*_X_s, *I*_syn_, and *I*_app_ represent currents through ion channels, synaptic currents, and the applied current, respectively. η_*i*_(*t*) represents Gaussian white noise. Because the GP neuron is inhibitory, the model neurons are connected by gamma-aminobutyric acid (GABA)ergic synapses. The kinetics of the synapses were described using the first-order kinetic model by Destexhe et al. (1998). The conductance of all synapses was set to 0.02 mS/cm^2^, which would amount to approximately 0.7–1.4 nS depending on the membrane area. The network model in the present study consisted of 64 GP neurons, each of which received GABAergic synaptic inputs from 8 randomly chosen GP neurons. Later in the analysis, where we saw how the proposed method performed for spike data from larger-size networks, the number of connections were set to 16 and 32 for the networks with 128 and 256 neurons, respectively. To maintain the similar firing rate, the maximal synaptic conductance was set to a half (0.01 mS/cm^2^) for the case of 128 neurons and a quarter (0.005 mS/cm^2^) for that of 256 neurons.

As reported in the previous study that prompted this research (Fujita et al., 2012), the set of the maximal conductances of the ion channels exerts a certain influence on the stable state of the GP network. Specifically, a combination of the maximal conductance values of persistent sodium channels (*g*_NaP_), Kv3 potassium channels (*g*_Kv3_), M-type potassium channels (*g*_KCNQ_), and calcium-dependent potassium channels (*g*_SK_) determines whether the network takes the monostable state of in-phase synchronization or the bistable state of in-phase and antiphase synchronization. We set the values of the four conductances according to two configurations: case A for the monostable in-phase synchronization state, and B for the bistable in-phase/antiphase state. For case A, *g*_NaP_ and *g*_Kv3_ were increased 20% from the reference values, whereas *g*_KCNQ_ and *g*_SK_ were decreased by 20%. For case B, the opposite changes in conductance were applied, rendering both in-phase and antiphase states stable. To equalize the firing rate of an isolated GP neuron for case A and B, it was necessary to set different values of applied current; *I*_app_ was set to 1.58 μA/cm^2^ for case A, and 2.74 μA/cm^2^ for case B, with the isolated firing rate adjusted to 40 spikes/s. In our simulation, the applied current for each neuron was drawn from a Gaussian distribution, *I*_app, i_ = *I*_app_(1 + ν_*i*_) where ν_*i*_ ~ *N*(0, $\sigma_{\text{I}}^{2}$), and where *N*(μ, σ^2^) denotes the density of a Gaussian distribution with the mean μ and the variance σ^2^. The standard deviation (σ_I_) was set to 10% of the value (σ_I_ = 0.1). Then, we tested the effect of increased firing rate variability by resetting the standard deviation to 30% of the value (σ_I_ = 0.0, 0.1, 0.2, and 0.3). This caused firing rates in the network to be distributed. η_*i*_(*t*) is independent Gaussian white noise drawn from the distribution *N*(0, $\sigma_{\text{N}}^{2}$). We here set σ_N_ to 0.0, 0.4, 0.8, and 1.6 μA/cm^2^. All other parameters were set as in the previous study that informed this research (Fujita et al., 2012).

### Phase response analysis

The dynamics of a limit cycle oscillator can generally be represented by a single degree of freedom, the phase. Furthermore, when such oscillators weakly interact with each other, the coupled limit cycle oscillators are described as coupled phase oscillators. In this phase description, the dynamics of the *i*th phase oscillator is

(2) $$\frac{d\phi_{i}}{dt}=\omega_{i}+\sum_{j\neq i}^{N}\Gamma_{ij}(\phi_{i}-\phi_{j})+\xi_{i}(t),$$

where ϕ_*i*_ and ω_*i*_ represent the phase and the natural frequency of the *i*th oscillator, respectively. We assumed that ξ_*i*_(*t*) was independent Gaussian white noise satisfying < ξ_*i*_(*t*)> = 0 and < ξ_*i*_(*t*) ξ_*j*_(*s*)> = 2*D*_*i*_δ_*ij*_δ(*t* – *s*). Γ_*ij*_(ϕ_*i*_ –ϕ_*j*_) is the interaction function from the *j*th oscillator to the *i*th oscillator, which can be theoretically derived as follows:

(3) $$\Gamma_{ij}(\Delta \phi_{ij})=\frac{1}{T}\int_{0}^{T}Z_{i}\left(\tau \right)I_{ij}\left(\tau ;\Delta \phi_{ij}\right)d\tau ,$$

where Δϕ_*ij*_, *T, Z*_*i*_(*t*), and *I*_*ij*_ (*t*; Δϕ_*ij*_) represent the phase difference between the *j*th and *i*th oscillator (Δϕ_*ij*_ = ϕ_*i*_–ϕ_*j*_), the period of oscillation, the phase response function of the receiver oscillator (the *i*th oscillator), and the input from the sender oscillator (the *j*th oscillator) to the receiver, respectively. The phase response function *Z*(*t*) represents the amount of phase advance (>0) or delay (<0) when an infinitesimal perturbation is given at *t*. Using the interaction function, the dynamics of the phase difference between two phase oscillators can be expressed as follows (Hansel et al., 1995):

(4) $$\frac{d\Delta \phi }{dt}=\Gamma_{\text{odd}}(\Delta \phi )\equiv \Gamma (\Delta \phi )-\Gamma (-\Delta \phi ).$$

Stable phase differences must satisfy the following equation:

(5) $$\Gamma_{odd}(\Delta \phi )=0\;and\;\frac{d\Gamma_{\text{odd}}(\Delta \phi )}{d\Delta \phi }<0$$

If Δϕ = 0 is a unique solution of Γ_odd_(Δϕ), the network of oscillators exhibits global synchrony. Therefore, to obtain the interaction function is essential to understanding the dynamic properties of the coupled oscillators.

When applying the method above to neural oscillators, we must obtain the phase response function *Z*(*t*) and the input between oscillators *I*(*t*). If the detailed dynamics of a neuron are known, it is possible to compute the phase response function *Z*(*t*) theoretically or numerically by solving the adjoint equation (Ermentrout, 1996; Nomura et al., 2003; Takekawa et al., 2007). In the present study, we numerically computed *Z*(*t*) from the neuronal dynamics of a GP neuron model, Equation (1). The inputs between neural oscillators were determined by the synapse model mentioned above (Destexhe et al., 1998). We computed the interaction functions for parameter sets A and B by using those functions.

In the network simulation, neurons received inhibitory synaptic inputs, which reduced their firing rates. The change in a firing rate (or a firing period) might cause a bifurcation of the solutions of Equation (5). Therefore, we confirmed the shapes of the interaction function for different firing rates or periods by computing the phase response function after altering the applied current. We then compared the estimated interaction function derived from simulated spike data (see *Bayesian estimation*) with the analytically derived function for the case of the same firing period as employed in the simulated spiking activity.

### Characterization of network states

We used the coherence measure to estimate the degree of synchrony in the neuronal population (Wang and Buzsáki, 1996). This measure was calculated for a pair of neurons *i* and *j* as follows:

(6) $$\kappa_{ij}=\frac{\sum_{n}x_{i}\left(t_{n}\right)x_{j}\left(t_{n}\right)}{\sqrt{\sum_{n}x_{i}(t_{n})\sum_{n}x_{j}(t_{n})}}$$

where *x*_*i*_(*t*_*n*_) represents a binary variable with 1 (a spike) or 0 (no spike) within the *n*-th bin for neuron *i*. We defined network state as the average coherence measure of all possible pairs in the network: κ = < κ_*ij*_ > _*ij*_. A change in the average coherence measure κ as a function of bin size characterizes a network state; a linear increase in the average coherence measure with an increase in bin size indicates uniform existence of spikes, i.e., an asynchronous firing pattern. However, the average coherence measure saturates with increased bin size if a neuronal population exhibits global synchronization, i.e., a synchronous firing pattern (Wang and Buzsáki, 1996).

### Bayesian estimation

Following the method previously proposed (Ota and Aoyagi, 2014), we used Bayesian estimation to determine ω_*i*_, Γ_*ij*_, and *D*_*i*_ in Equation (2). Because the interaction function Γ_*ij*_ is a nonlinear function, it is generally difficult to estimate. However, the Bayesian approach allows us to estimate the function, and consequently the phase dynamics, successfully from time-series data.

To obtain phase time-series data from the observed spike data, the proposed method required modification. Spike data provide only information regarding timing when phase variables have specific values and do not contain detailed information on temporal changes in phase variables. Here we defined a spike time as zero of a phase variable, i.e., ϕ(*t*_spk_) = 0, where *t*_spk_ denotes the time of a spike. Because ω_*i*_ is generally very large compared to Γ_*ij*_ (Δϕ_*ij*_) and ξ_*i*_(*t*), we linearly approximated temporal changes in phase variables and interpolated phase values between the *s*th and the (*s* + 1)th spikes as follows:

(7) $$\phi (\tau )=2\pi \frac{\Delta t}{t_{s+1}-t_{s}}\tau \;\left(\tau =1,2,\ldots ,T_{s}-1\right),$$

where *t*_*s*_ denotes the time of the *s*th spike of a neuron. The constant Δ*t* is a sampling interval that determines the number of intervals *T*_*s*_ as follows: *T*_*s*_ = (*t*_*s*__+1_ – *t*_*s*_)/Δ*t*.

We required some other parameters to describe the function Γ_*ij*_ (Δϕ_*ij*_). Because the interaction function is a 2π-periodic function, it can be expanded in a Fourier series as follows:

(8) $$\Gamma_{ij}(\Delta \phi_{ij})=a_{ij}^{\left(0\right)}+\sum_{m=1}^{M_{i}}\left[a_{ij}^{\left(m\right)}\text{cos}\left(m\Delta \phi_{ij}\right)+b_{ij}^{\left(m\right)}\text{sin}\left(m\Delta \phi_{ij}\right)\right],$$

where the variable *M*_*i*_ denotes the number of harmonics for each Γ_*ij*_ (Δϕ_*ij*_) and controls the model complexity. Using these notations, we rewrote the Model Equation (2) as follows:

(9) $$\begin{array}{l} \frac{d\phi_{i}}{dt}={\hat{\omega }}_{i}+\sum_{j\neq i}^{N}\sum_{m=1}^{M_{i}}\left[a_{ij}^{\left(m\right)}\text{cos}\left(m\Delta \phi_{ij}\right)+b_{ij}^{\left(m\right)}\text{sin}\left(m\Delta \phi_{ij}\right)\right]\\ \;+\xi_{i}(t), \end{array}$$

where we treated ${\hat{\omega }}_{i}\equiv \omega_{i}+\sum_{j\neq i}^{N}a_{ij}^{\left(0\right)}$ as a single parameter because the contributions of ω_*i*_ and *a*_*ij*_^(0)^ to the dynamics are inseparable. Thus, the dynamics are estimated by evaluating 2+2*M*_*i*_(*N*−1) unknown parameters, ${\hat{\omega }}_{i}$, *D*_*i*_, and {*a*_*ij*_^(m)^, *b*_*ij*_^(m)^}_m, *j*_. For simplicity, hereafter we use the shorthand notations ${\text{c}}_{i}\equiv {\left[{\hat{\omega }}_{i},{\text{c}}_{i,1},\ldots ,{\text{c}}_{i,i-1},{\text{c}}_{i,i+1},\ldots ,{\text{c}}_{i,N}\right]}^{T}$ with

(10) $$c_{i,j}\equiv [a_{ij}^{\left(1\right)},b_{ij}^{\left(1\right)},a_{ij}^{\left(2\right)},b_{ij}^{\left(2\right)},\ldots ,a_{ij}^{\left(M_{i}\right)},b_{ij}^{\left(M_{i}\right)}]$$

and

(11) $${\hat{\Gamma }}_{ij}(\Delta \phi_{ij})\equiv \sum_{m=1}^{M_{i}}\left[a_{ij}^{\left(m\right)}\text{cos}(m\Delta \phi_{ij})+b_{ij}^{\left(m\right)}\text{sin}(m\Delta \phi_{ij})\right].$$

We next evaluated all unknown parameters to describe the phase dynamics following the standard Bayesian approach (Bishop, 2006; Murphy, 2012). In this approach, when we obtain new observed data {ϕ_*i*_(*t*)}, the parameter distribution *p*(**c**_*i*_, *D*_*i*_) is updated as follows:

(12) $$p(c_{i},D_{i}|\{\phi_{i}(t)\})\propto p(\{\phi_{i}(t)\}|c_{i},D_{i})p(c_{i},D_{i}),$$

where *p*({ϕ_*i*_(*t*)}|**c**_*i*_, *D*_*i*_) is the likelihood function, which denotes the probability of reproducing the observed data for the given parameters. For this update, we needed to determine the likelihood function, initial distribution of the parameters, and model complexity.

First, we defined the likelihood function. It is by virtue of this function that phase time-series data {ϕ_*i*_(*t*)}, including interpolated data sampled at *T* equal intervals, can be obtained. The likelihood function in our method was defined as follows:

(13) $$\begin{array}{l} p(\{\phi_{i}(t)\}|c_{i},D_{i})\\ \;=\prod_{\tau =0}^{T-1}N\left(\frac{\phi_{i}(\tau +1)-\phi_{i}(\tau )}{\Delta t}|{\hat{\omega }}_{i}+\sum_{j\neq i}^{N}{\hat{\Gamma }}_{ij}(\Delta \phi_{ij}),\frac{2D_{i}}{\Delta t}\right) \end{array}$$

The phase variables ϕ_*i*_(0) and ϕ_*i*_(*T*) take values of 0 and 2π, respectively.

Next, we used a conjugate prior distribution as the initial distribution so that the posterior distribution had the same functional form as the prior distribution. This enabled us to derive the posterior distribution by only updating the values of hyperparameters that characterize the conjugate prior distribution. In our approach, we adopted a Gaussian-inverse-gamma distribution, as follows:

(14) $$p(c_{i},D_{i})\propto \text{exp}\left(-\frac{{\left(c_{i}-\chi_{i}\right)}^{T}\Sigma_{i}^{-1}(c_{i}-\chi_{i})+2\beta_{i}}{2\sigma_{i}^{2}}\right){\left(\sigma_{i}^{2}\right)}^{-\frac{P_{i}}{2}-\alpha_{i}-1}$$

where *P*_*i*_ is the dimension of the vector **c**_*i*_. The variable σ_*i*_^2^ is defined as $\sigma_{i}^{2}\equiv 2D_{i}/\Delta t$. **χ**_*i*_, Σ_*i*_, α_*i*_, and β_*i*_ denote hyperparameters. Thus, we obtained the posterior distribution after updating the hyperparameters:

(15) $$\begin{array}{l} \chi_{i}^{\text{new}}=\Sigma_{i}^{\text{new}}(F_{i}^{T}\delta_{i}+{\left(\Sigma_{i}^{\text{old}}\right)}^{-1}\chi_{i}^{\text{old}}),\\ \Sigma_{i}^{\text{new}}={\left\{{\left(\Sigma_{i}^{\text{old}}\right)}^{-1}+F_{i}^{T}F_{i}\right\}}^{-1},\\ \alpha_{i}^{\text{new}}=\alpha_{i}^{\text{old}}+\frac{T}{2},\\ \beta_{i}^{\text{new}}=\beta_{i}^{\text{old}}+\frac{1}{2}\{\delta_{i}^{T}\delta_{i}+{\left(\chi_{i}^{\text{old}}\right)}^{T}{\left(\Sigma_{i}^{\text{old}}\right)}^{-1}\chi_{i}^{\text{old}}\\ \;-{\left(\chi_{i}^{\text{new}}\right)}^{T}{\left(\Sigma_{i}^{\text{new}}\right)}^{-1}\}. \end{array}$$

Here, we defined *T-*dimensional column vectors, (δ_*i*_)_τ_ = {ϕ_*i*_(τ+1)– ϕ_*i*_(τ)}/ Δ*t* (τ = 0, 1,…, *T*−1), and *T* × {1+2*M*_*i*_(*N*−1)} matrices

(16) $$F_{i}=\left[\begin{array}{ccccccc} 1 & G_{i,1}^{0} & \ldots & G_{i,i-1}^{0} & G_{i,i+1}^{0} & \cdots & G_{i,N}^{0}\\ \vdots & \vdots & & \vdots & \vdots & & \vdots \\ 1 & G_{i,1}^{T-1} & \cdots & G_{i,i-1}^{T-1} & G_{i,i+1}^{T-1} & \cdots & G_{i,N}^{T-1} \end{array}\right]$$

with *M*_*i*_-dimensional row vectors, where $G_{i}{}_{j}^{\tau }$ was defined as *G*_*i*_,${}_{j}^{\tau }$ = [cosΔϕ_*ij*_(τ), sinΔϕ_*ij*_(τ), cos(2Δϕ_*ij*_(τ)), sin(2Δϕ_*ij*_(τ)), ···, cos(*M*_*i*_Δϕ_*ij*_(τ)), sin(*M*_*i*_Δϕ_*ij*_(τ))]. The superscripts “new” and “old” indicate the hyperparameters of the posterior and prior distributions, respectively.

Finally, we determined the model complexity *M*_*i*_. Following the Bayesian model selection method, we determined the value of *M*_*i*_ by maximizing the model evidence given by the following equation:

(17) $$p(\{\phi (t)\}|M_{i})=\frac{p(\{\phi (t)\}|c_{i},D_{i})p(c_{i},D_{i})}{p(c_{i},D_{i}|\{\phi_{i}(t)\})}.$$

Because it is generally impossible to analytically optimize model evidence, we explored the optimal parameter value within the range of 1–5. We calculated the model evidence for each of these values and then chose the value that resulted in the largest model evidence as the optimal value.

### Evaluation of estimation performance

To evaluate estimation performance, we calculated the L^2^-distance between two vectors ${\text{c}}_{\text{i},\text{j}}^{*}$ and **c**_i, *j*_, which characterized the theoretical function ${\hat{\Gamma }}_{ij}^{*}\left(\Delta \phi_{ij};{\text{c}}_{i,j}^{*}\right)$ and the estimated function ${\hat{\Gamma }}_{ij}$(Δφ_*ij*_; **c**_*i,j*_), respectively. The L^2^-distance was given by the following equation:

(18) $$d_{ij}\left(c_{i,j}^{*},c_{i.j}\right)\equiv \sqrt{\sum_{k=1}^{2M_{i}}{\left(c_{k}^{*}-c_{k}\right)}^{2}+\sum_{l=2M_{i}+1}^{2n}{\left(c_{l}^{*}-0\right)}^{2}}$$

where {*c*_k_^*^} and {*c*_*k*_} denote ${\text{c}}_{\text{i},\text{j}}^{*}$ and **c**_*i, j*_, respectively. The constant *n* is sufficiently larger than *M*_*i*._

### Inference of synaptic connections

We inferred synaptic connections between neurons on the basis of the magnitudes of the estimated interaction functions in order to evaluate the performance of the estimation from another viewpoint. Using Otsu's method (Otsu, 1979), we estimated the threshold and regarded a synapse as connected (*w*_*ij*_^est^ = 1) if the magnitude of the corresponding interaction function was greater than the threshold; otherwise, synapses were considered unconnected (*w*_*ij*_^est^ = 0).

We quantitatively compared the inferred synaptic connections (*w*_*ij*_^est^) with the actual connections in the network model (*w*_*ij*_^act^) using Matthew's correlation coefficient (MCC), which is a measure of classification quality (Baldi et al., 2000; Kobayashi and Kitano, 2013). The case in which both connections exist for the *j*th to *i*th neuron (*w*_*ij*_^est^ = 1 and *w*_*ij*_^act^ = 1) is regarded as a True Positive (TP). Cases with (*w*_*ij*_^est^, *w*_*ij*_^act^) = (1, 0), (0, 0), and (0, 1) are called a False Positive (FP), a True Negative (TN), and a False Negative (FN), respectively. After obtaining the numbers of these four types of results for the neuron pairs, we calculated the MCC using the equation below:

(19) $$MCC=\frac{TP\cdot TN-FP\cdot FN}{\sqrt{\left(TP+FP)(TP+FN))(TN+FP)(TN+FN\right)}}.$$

The MCC becomes 1 if the two classes (*w*_*ij*_^est^ and *w*_*ij*_^act^) match perfectly, while a random guess results in a value of approximately 0.

## Results

**Figure 2:**
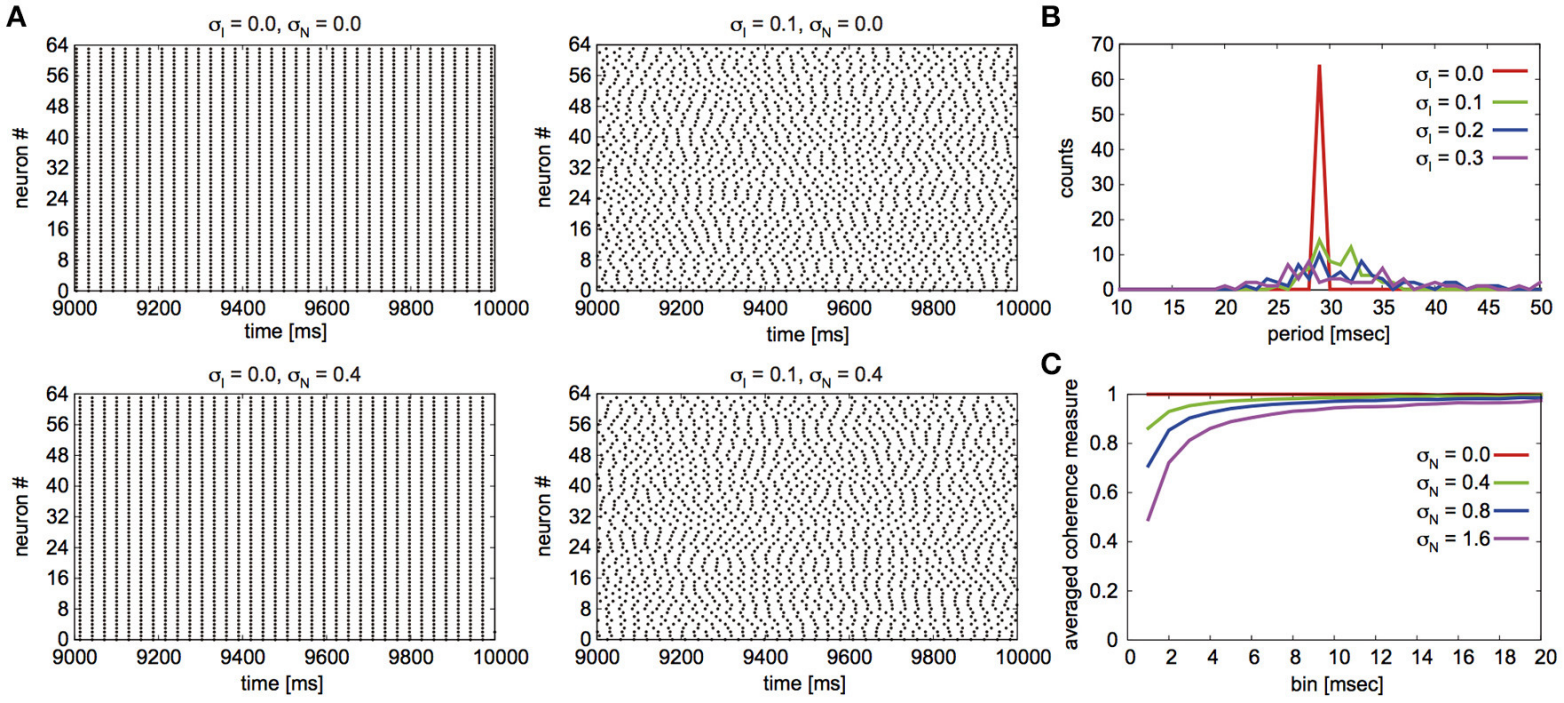
Simulated neuronal spikes that were utilized in the phase dynamics estimation. Raster displays of a globus pallidus neuronal network and characteristics of the network activity for case A. Spiking activity of the neurons under the various conditions of the variation in applied current and noise **(A)**, distributions of firing periods for the different standard deviations σ_I_ when σ_N_ was set to 0.4 **(B)**, and changes in average coherence measure as a function of bin size for different standard deviations of noise σ_N_ when σ_I_ = 0 **(C)**.

**Figure 3:**
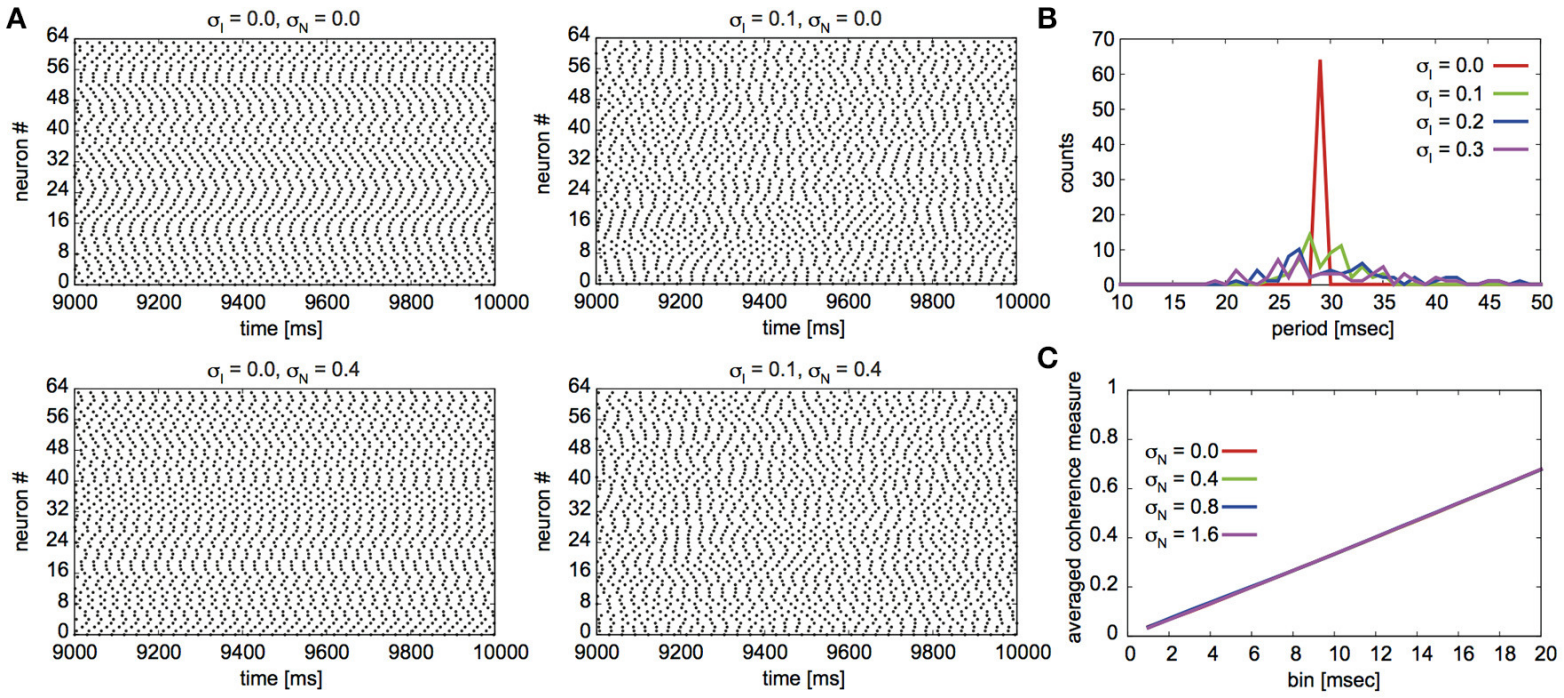
Simulated neuronal spikes utilized in the phase dynamics estimation. Raster displays of a globus pallidus neuronal network and characteristics of the network activity for case B. Spiking activity of the neurons under the various conditions **(A)**, distributions of firing periods for the different standard deviations σ_I_ **(B)**, and changes in the average coherence measure as a function of bin size for different standard deviations of noise σ_N_ **(C)**. The parameter values are the same as those described in Figure 2.

**Figure 4:**
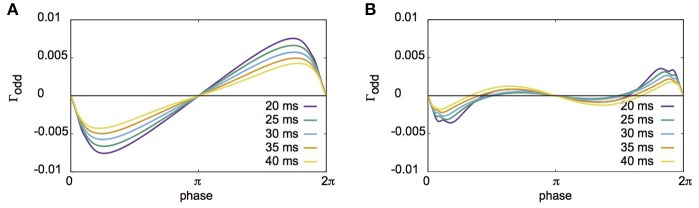
The odd parts of interaction functions derived from the phase response analysis for different firing periods. **(A)** The odd parts of interaction functions for case A. For all periods investigated here, the stable phase difference was only 0 (or 2π). **(B)** Similar plot to A, but for case B. The stable phase differences in this case were 0 and π, suggesting that coupled neurons had bistable states of in-phase and antiphase synchronization.

Fujita et al. (2012) reported their network to exhibit two distinct states for different parameter sets: global synchrony (case A) and two clusters corresponding to in-phase and antiphase synchrony (case B). Figures 2, 3 display the firing patterns of the network model for various conditions for cases A and B, respectively. Figure 2A is the raster display of the neurons in the network under the conditions of case A for different conditions of σ_I_ and σ_N_. When all the neurons exhibited the same firing rate or period (σ_I_ = 0), the network globally synchronized irrespective of the presence of noise. In contrast, when the firing periods varied (σ_I_ = 0.1), the neurons no longer showed global synchronization. As shown in Figure 2B, firing periods were distributed on the basis of different values of σ_I_ (σ_I_ = 0.0: 29.03 ± 0.00 [mean ± standard deviation] ms; 0.1: 31.10 ± 2.32 ms; 0.2: 32.32 ± 5.32 ms; 0.3: 34.37 ± 9.80 ms). Figure 2C illustrates the changes in average coherence measure with or without noise as a function of bin size (σ_I_ = 0). Although the precision of synchronization reduced with an increase in noise intensity, the neurons synchronized even with increased noise intensity. The network activity patterns for case B are displayed in Figure 3A. When the firing periods equalized, neuronal spikes phase-locked with finite phases irrespective of noise. However, such phase-locking was lost when the firing periods varied as shown in Figure 3B (σ_I_ = 0.0: 29.40 ± 0.05 ms; 0.1: 30.08 ± 2.49 ms; 0.2: 31.55 ± 5.95 ms; 0.3: 34.37 ± 12.07 ms). The changes in the average coherence measure increased linearly, suggesting that the spikes were uniformly distributed (Figure 3C). As shown in Figures 2B, 3B, increased variance in the applied current changed the neuronal firing periods. Since a change in the firing period can yield different stable solutions, we confirmed the stability of the phase differences by applying the phase response analysis to different firing periods (see Materials and Methods). Figures 4A,B illustrate the odd parts of the interaction functions for cases A and B, respectively. For case A, the stable phase difference was only 0, indicating that only in-phase synchrony is stable for the examined firing periods. For case B, both 0 and π were the stable phase differences, suggesting that in-phase and antiphase synchrony are bistable. In both cases, stable phase differences were not affected by changes in firing period.

**Figure 5:**
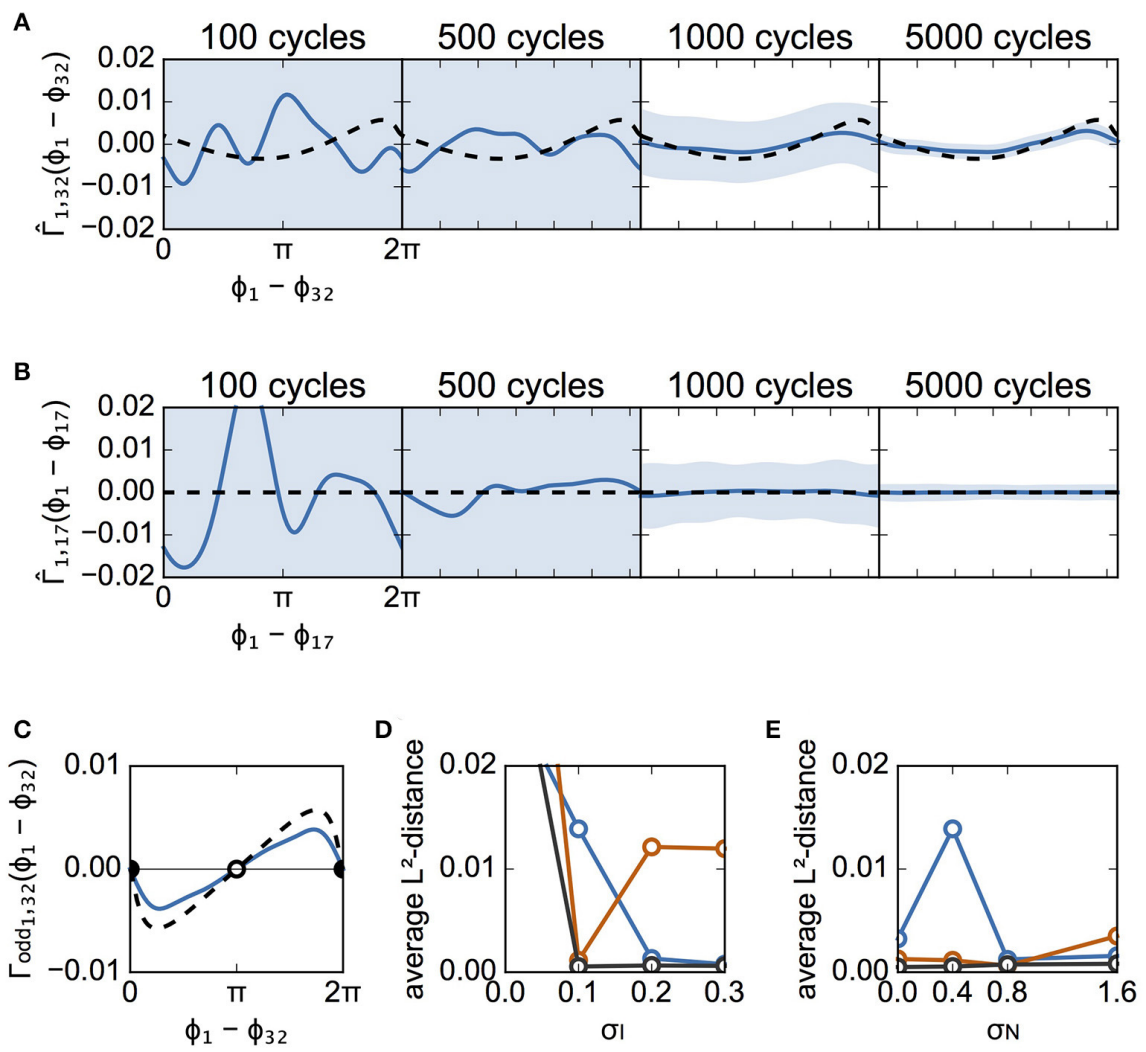
Estimated interaction function for case A. **(A)** Estimated interaction functions $\hat{\Gamma }\left(\Delta \phi \right)$ of a connected neuron pair for various cycle numbers of data (blue lines). $\hat{\Gamma }\left(\Delta \phi \right)$ derived from the detailed model is also shown for comparison (dashed lines). The light blue zone represents the 95% confidence interval. The vertical scale is in rad/ms. The L^2^-distances were 0.0107, 0.0066, 0.0023, and 0.0021, for 100, 500, 1,000, and 5,000 cycles, respectively. **(B)** Similar to **(A)**, except that this panel describes the results from an unconnected neuron pair. Since there was no interaction function for this neuron pair, the theoretically derived $\hat{\Gamma }\left(\Delta \phi \right)$ is represented by the flat dashed lines. The L^2^-distances were 0.0490, 0.0117, 0.0014, and 0.0002, for 100, 500, 1,000, and 5,000 cycles, respectively. **(C)** Comparison of the odd part of $\hat{\Gamma }\left(\Delta \phi \right)$ between the estimated and theoretically obtained functions. The filled circles indicate stable phase differences whereas the open circle shows an unstable phase difference. Although a discrepancy between the odd parts is seen, they have the same stable phase difference (Δϕ = 0). **(D)** Dependence of L^2^-distances on the variation of applied currents. The averages of L^2^-distance *d*_1j_ (*j* = 2, 3, …, 64) were calculated with several numbers of cycles. Blue, red, and black lines indicate the results for 500, 1,000, and 5,000 cycles, respectively. σ_N_ was set to 0.4. **(E)** Dependence of L^2^-distances on the standard deviation of noise. The usage of colors is the same as in **(D)**. σ_I_ was set to 0.1.

We applied the Bayesian approach to the spike data to estimate the interaction functions between the neuron pairs. Figure 5 shows the estimated interaction functions between a connected pair and an unconnected pair in case A. Figure 5A shows the estimated interaction function from neuron #32 to neuron #1 for several different numbers of spike cycles. Because there was a synaptic connection in that direction between the neurons, the interaction function theoretically obtained from the detailed model is indicated in Figure 5A by a dashed line. As more data were used, the estimated function took a shape more analogous to the theoretically derived function. In contrast, the network had no synapse to transmit a signal from neuron #17 to neuron #1. There was thus no interaction function in that direction. The estimated function thus took on a flat shape when 1,000 cycles of spike data were used. This confirms the estimation of the unconnected synapse to have been correct (Figure 5B). The shape of the interaction function determines the stability of the network states. Because the odd part of the function, Γ_odd_, describes the stable states, we compared Γ_odd_ of the estimated function with that of the theoretically obtained function in Figure 5C. Although the scales were different, the estimated function yielded the same stable phase difference (Δϕ = 0) as the theoretical function. Thus, if the data of a sufficient number of spike cycles are available, the Bayesian approach can successfully estimate the interaction function with the parameters of case A. Estimated functions were evaluated by L^2^-distance between theoretically derived and experimentally derived interaction functions for various parameters (see section Materials and Methods). First, we determined the dependence of the distance on the magnitude of σ_I_ for σ_N_ = 0.4 (Figure 5D). All distances except for σ_I_ = 0.0 were improved as the data lengths (cycles) became larger. When σ_I_ = 0.0, the distance was large for all data lengths, which indicates that the proposed method did not perform well in this case. This poor estimation was independent of whether a neuron pair had direct connections (Figure S1). Figure 5E shows the dependence of the distance on the magnitude of σ_N_ for σ_I_ = 0.1. It is suggested that the distance was not significantly affected by σ_N_ when compared to σ_I_. In this case, the distance increased as σ_N_ increased (red, 1,000 cycles).

**Figure 6:**
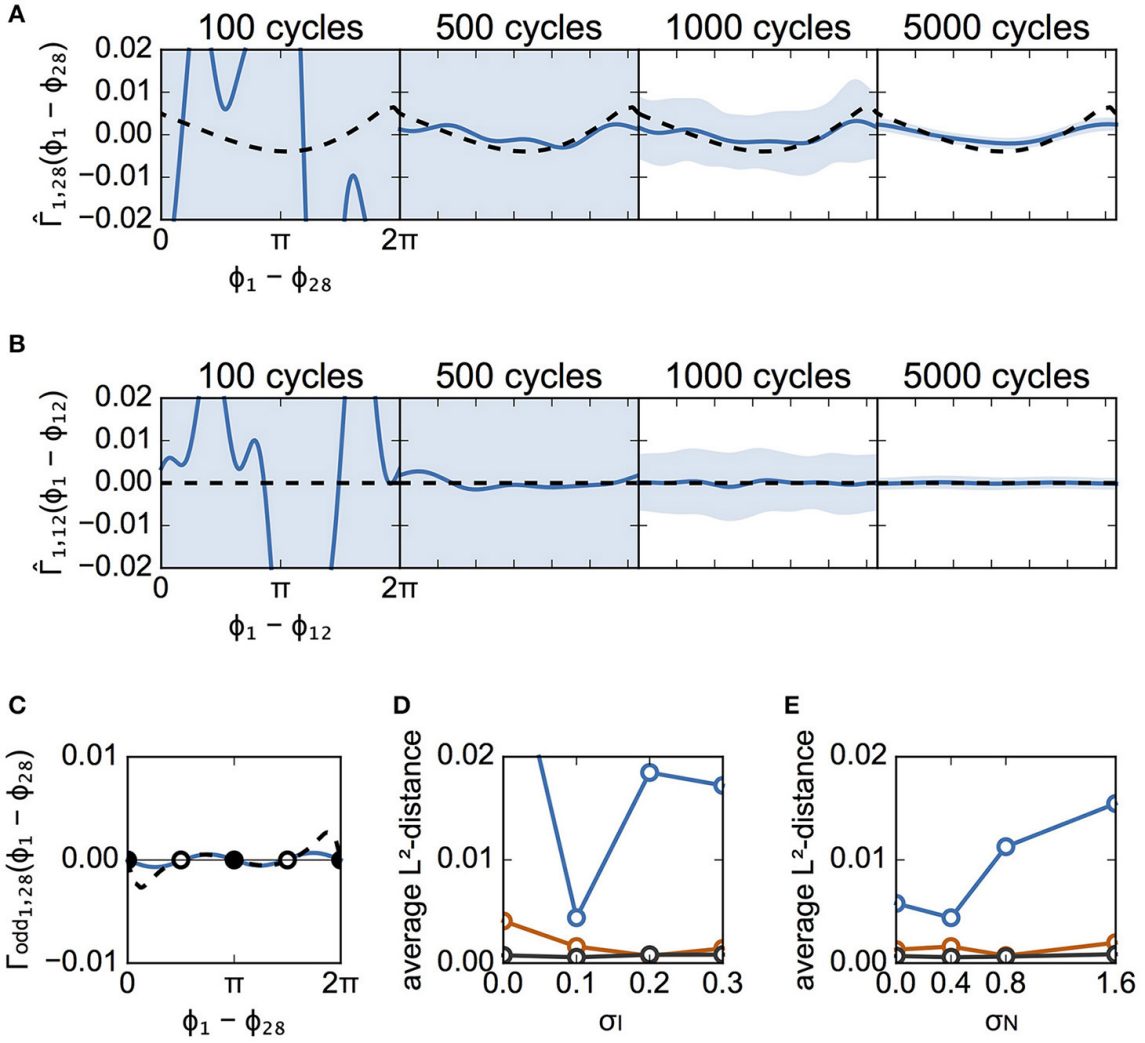
Estimated interaction function for case B. This figure is similar to Figure 5, except that it describes the results from case B instead of case A. **(A)** The estimated interaction functions of a connected neuron pair for various cycle numbers of data (blue lines). The light blue zone represents the 95% confidence interval. $\hat{\Gamma }\left(\Delta \phi \right)$ derived from the detailed model is also shown for comparison (dashed lines). The L^2^-distances were 0.1235, 0.0044, 0.0016, and 0.0002, for 100, 500, 1,000, and 5,000 cycles, respectively. **(B)** The estimated interaction functions of an unconnected neuron pair. The L^2^-distances were 0.1261, 0.0062, 0.0015, and 0.0003, for 100, 500, 1,000, and 5,000 cycles, respectively. **(C)** Comparison of the odd part of $\hat{\Gamma }\left(\Delta \phi \right)$ between the estimated and theoretically obtained functions. As in Figure 5C, the filled circles indicate stable phase differences while the open circles show unstable phase differences. **(D,E)** Dependence of L^2^-distances on the standard deviation of applied currents and of noise. σ_N_ was set to 0.4 for **(D)**, while σ_I_ was set to 0.1 for **(E)**.

Figure 6 is similar to Figure 5, except that it describes case B instead of case A. Figures 6A,B illustrate the estimated interaction functions of a connected neuronal pair (from #28 to #1) and an unconnected pair (from #12 to #1), respectively. In addition, so long as data with a sufficient number of spike cycles were used in case B, the estimated interaction functions took on the expected shape (i.e., analogous to the shape of the theoretical functions for a connected pair or a flat shape for an unconnected pair). Similar to case A, although the estimated Γ_odd_ was different from the theoretical one, the stabilities of the two solutions for phase differences 0 and π were unchanged (Figure 6C). Similar to Figures 5D,E,6D,E show the dependence of the L^2^-distances on σ_I_ and σ_N_, respectively. As shown in Figure 6D, except for the case with 500 cycles, the distances for all values of σ_I_ were smaller than those in case A (Figure 5D). Even when σ_I_ = 0.0, the phase differences were distributed (Figures 3A,C) due to random connections, which enabled us to collect information regarding the phase relations utilized for reconstruction of the interaction functions (data not shown). The distance increased as σ_N_ became larger in case B as well.

**Figure 7:**
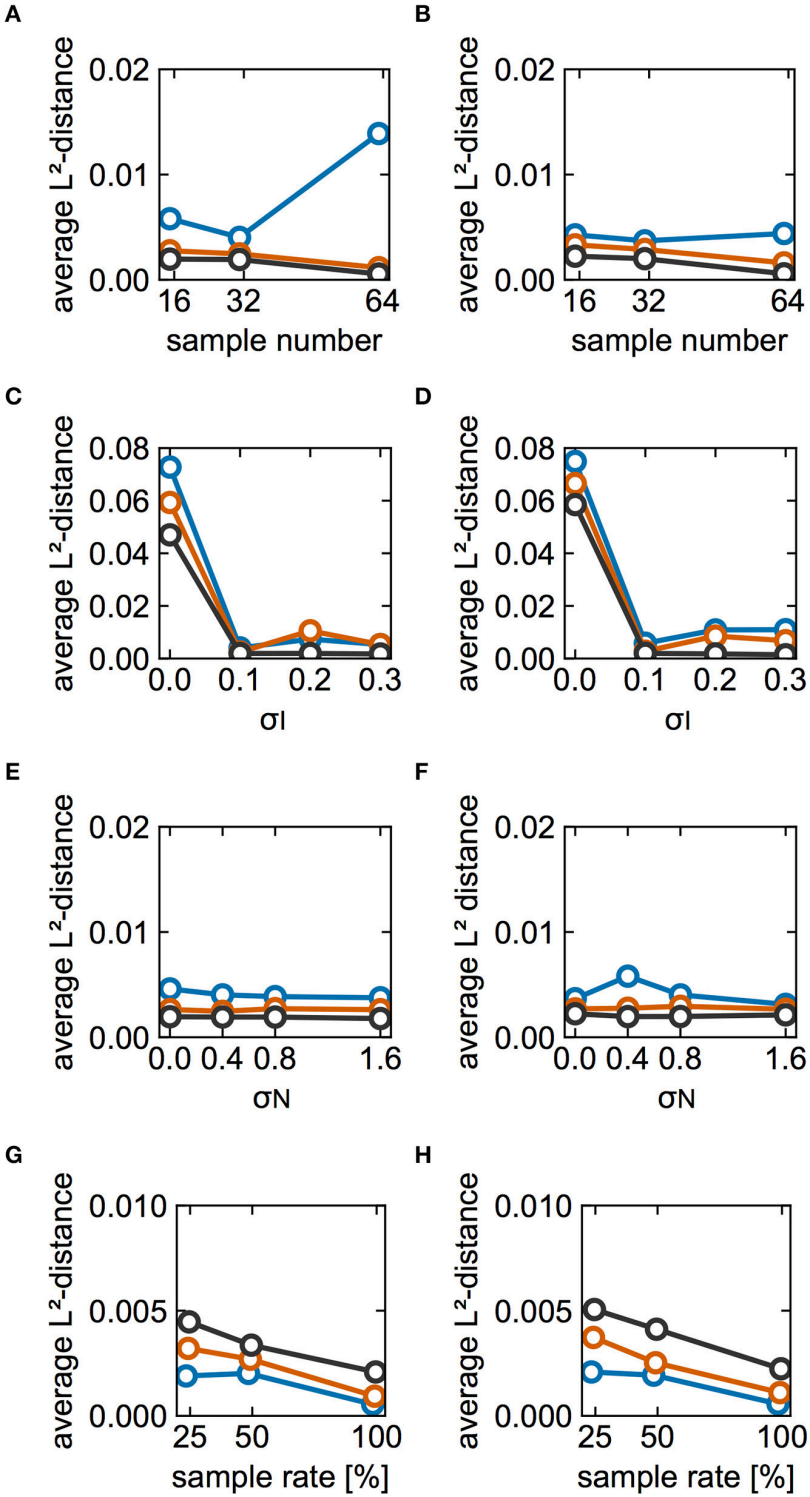
Effects of partial observation on estimation performance. **(A,B)** Dependence of the L^2^-distances on the numbers of sampled neurons for cases A and B, respectively. Here we plotted L^2^-distances averaged over neuron pairs between the reference neuron (#1) and other sampled neurons for 20 different sampling runs. Blue, red, and black lines indicate the results for 500, 1,000, and 5,000 cycles, respectively. The color used here are consistent with those used elsewhere in this figure. σ_I_ and σ_N_ were set to 0.1 and 0.4, respectively. **(C**,**D)** Dependence of the average L^2^-distance on different values of σ_I_. **(C,D)** Display the results for cases with 32 and 16 sampled neurons, respectively. σ_N_ was set to 0.4. **(E,F)** Dependence of the averaged L^2^-distance on different values of σ_N_. Similar to **(C–F)** display the results for cases with 32 and 16 sampled neurons, respectively. σ_I_ was set to 0.1. **(G**,**H)** Dependence of the average L^2^-distance on the size of network and the number of sampled neurons for case A and B, respectively. For comparison, the average L^2^-distances were scaled by the relative synaptic strengths, i.e., the distances for the 128-neuron network were doubled whereas those for the 256-neuron network were quadrupled. The sample rate 25, 50, and 100% correspond to 32, 64, and 128 neurons for the 128-neuron network whereas 64, 128, and 256 neurons for the 256-neuron network. Blue, red, and black lines indicate the results for the 64-, 128-, and 256-neuron networks, respectively. The number of cycles and trials were 5,000 and 10, respectively. σ_I_ and σ_N_ were set to 0.1 and 0.4, respectively.

The effect of partial observation on estimation performance was investigated. In the above analyses, we utilized spike data from all of the neurons in the model. Here, the results pertain to spikes recorded from a subset of modeled neurons. Figures 7A,B show the L^2^-distance averaged over the sampled neurons as a function of the number of sampled neurons for cases A and B, respectively. The estimation performance deteriorated with decreasing numbers of sampled neurons. Figures 7C,D illustrate how the averaged distances depended on σ_I_ when the numbers of sampled neurons were 32 and 16, respectively. Although the distance depends highly on both the number of cycles and σ_I_, estimation performance for the case with 32 neurons was better than that of the case with 16 neurons for all values of σ_I_ when sufficient data were available (black, 5,000 cycles). Similarly, Figures 7E,F illustrate the dependence on σ_N_ in the cases with 32 and 16 neurons, respectively. In these analyses, the average distances for 32 neurons were slightly smaller than those for 16 neurons when we considered 5,000 cycles of spike data. In this case, the estimation result did not depend on whether a neuron pair had a direct connection. Subsequently, we examined whether the dependence of the estimation performance on partial sampling was the case for larger-sized networks. We increased the number of neurons in the network to 128 and 256. With an increase in the network size, the number of connections was increased whereas the synaptic strengths were decreased (see section Materials and Methods for details). Under this condition, the firing periods in the network of 128 neurons exhibited 30.94 ± 2.68 ms for case A and 29.87 ± 2.91 ms for case B. Similarly, those in the network of 256 neurons were 30.95 ± 2.27 ms for case A and 29.89 ± 2.42 ms for case B. Figures 7G,H demonstrate how the L^2^-distances depended on the network size for cases A and B, respectively. Similar to the case of the 64-neuron network, the estimation deteriorated as the number of sampled neurons (or the sample rate) was reduced. Furthermore, as the network size was increased, the L^2^-distances scaled by the synaptic strengths were increased, suggesting that the estimation performance deteriorated. This is because the synaptic strengths were decreased for the larger-size networks and it was harder for the proposed method to detect changes in phase differences for such a case.

**Figure 8:**
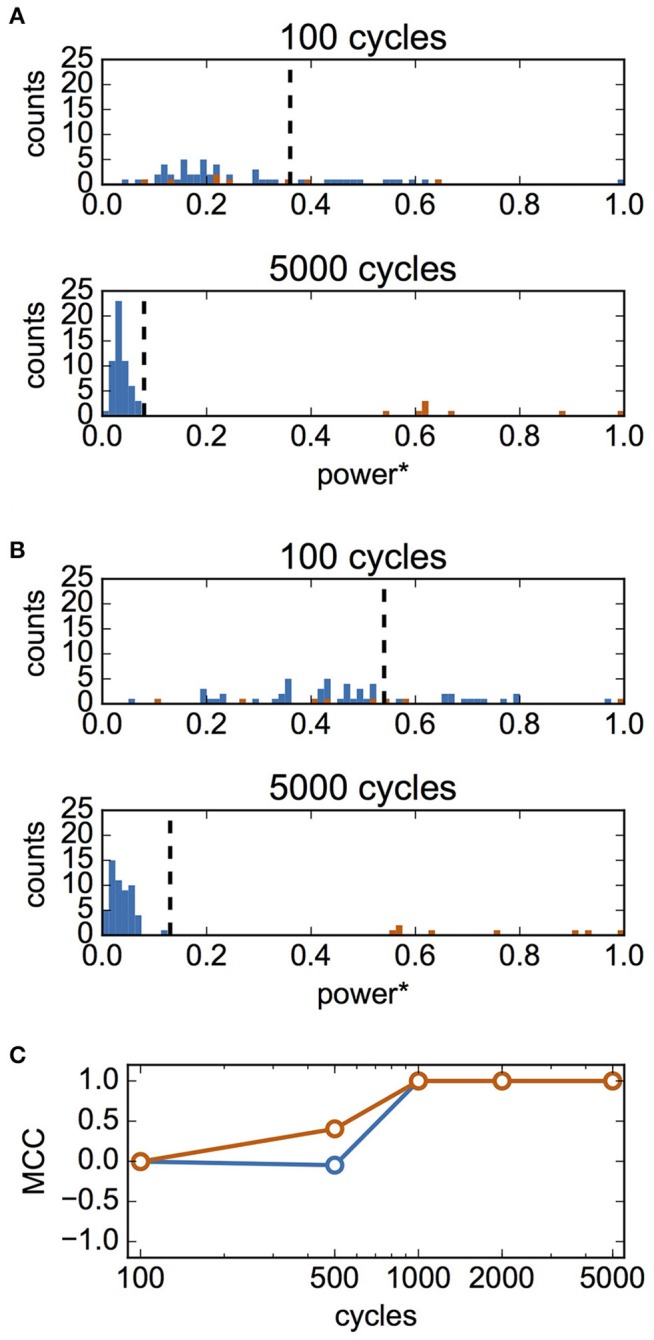
Inference of synaptic connections based on estimated interaction functions. In this analysis, σ_I_ and σ_N_ were set to 0.1 and 0.4, respectively. **(A)** Distributions of summed powers of $\hat{\Gamma }\left(\Delta \phi \right)$ for case A. The values were normalized using the maximum value, max_*j*_(*P*_1j_). The upper panel shows the result for 100-cycle data, and the bottom panel shows that for 5,000-cycle data. Blue and red histograms display the distributions of unconnected neuron pairs and connected neuron pairs, respectively. The vertical dashed lines show the thresholds determined using Otsu's method. These lines were used as the thresholds for inference of the presence of synaptic connections. **(B)** Distributions of summed powers for case B. As in **(A)**, the upper panel shows the result for 100-cycle data, and bottom panel shows that for 5,000-cycle data. **(C)** Matthew's correlation coefficient (MCC) for the inference of synaptic connections as a function of cycle number. Blue and red indicate MCC for case A and B, respectively.

Finally, we examined whether it is possible to infer synaptic connections based on the estimated interaction functions. We hypothesized that the summed powers of the Fourier coefficients of the estimated interaction function $\sum_{m=1}^{M_{i}}\left\{{\left(a_{ij}^{\left(m\right)}\right)}^{2}+{\left(b_{ij}^{\left(m\right)}\right)}^{2}\right\}$ would be a good criterion for the presence of a synaptic connection. We calculated the normalized summed powers of the function *P*_1j_ for 63 pairs of neurons (from neuron #*j* to neuron #1, *j* = 2, 3,…, 64). We then applied the Otsu method to these values to classify them into two clusters based on the threshold. When *P*_1j_ was larger than the threshold, it was inferred that a synaptic connection was present from neuron #*j* to #1. Figure 8A shows the distribution of *P*_1j_ for case A. The top and bottom panels of Figure 8A show the results of fitting for 100- and 5,000-cycle spike data, respectively. The summed powers of neuron pairs with (red) and without (blue) actual connections were merged for 100-cycle data, but were clearly separated for 5,000-cycle data. In Figure 8B, the distributions of the summed powers for case B are shown. These data suggest that 100-cycle spike data were not sufficient for the inference of synaptic connections, while 5,000-cycle spike data were useful for this purpose. The correlation between actual and inferred connections was evaluated using MCC. In Figure 8C, MCC values are shown as functions of cycle number. Although the inferred connections did not correlate with actual connections for 100-cycle data, our method worked perfectly for 1,000-cycle data.

## Discussion

Constructing a detailed dynamic model based on measured data is often difficult and involves the selection of an appropriate model, adjustment for many unknown parameters, and insufficient information regarding measured data used for modeling. Furthermore, incorrect modeling can lead to misunderstanding of the properties of the dynamic system. For cases wherein the dynamic system consisted of nonlinear oscillators, Ota and Aoyagi (2014) have proposed a Bayesian method through which the reduced dynamics of a complicated dynamic system can be derived directly based on measured data (Tokuda et al., 2007; Kralemann et al., 2008; Cadieu and Koepsell, 2010; Stankovski et al., 2012). This approach is based on the fact that such oscillator systems can be represented by the reduced dynamics of phase oscillators (Ermentrout and Kopell, 1984; Kuramoto, 1984; Hansel et al., 1995). The method requires time-series data to construct the phase dynamics because the time-series data contain detailed phase information of oscillatory units. This method would be a powerful tool for the analysis of experiments recoding time-series data such as that of the electroencephalogram. However, it is still difficult to record time-series data, such as membrane potentials, from multiple neurons to investigate neural activity.

To apply the Bayesian method to spike data, it is necessary to interpolate phase values between spikes because spike timing only provides a specific phase value. Because no detailed information regarding the interspike phase values was available, we simply employed linear interpolation. This simple approximation method can be used to extract only a limited amount of information regarding the dynamics of each cycle. In addition, the neuronal network model used in the present study was rather complex and included 10 different ionic currents. Nevertheless, we were also able to use the Bayesian method to estimate the interaction function for the different conditions leading to the different network states (Figures 5, 6). Additionally, using the estimated interaction functions to infer the synaptic connections worked perfectly, although this was the case only when spike data with high cycle numbers were used (Figure 8). The method performed poorly when the network exhibited global synchronization (Figure 5 and Figure S1), when low cycle number spike data were used (Figures 5–8), when the fraction of recorded neurons was small (Figure 7), and when the synaptic strengths were weak (Figure 7). Thus, even though the data available to extract the phase dynamics consisted only of spikes, the effectiveness of the method was confirmed for several different conditions.

Estimation performance depended on firing period variability, noise intensity, and the number of sampled neurons. Although a higher degree of noise tended to degrade the estimation performance, noise intensity had a limited impact on estimation performance relative to the other factors when sufficient spike data (>5,000 cycles) were available. To examine the *in vivo* firing rate of GP neurons, which ranges from 30 to 50 spikes/s, a much greater input than the threshold current was applied. This resulted in a modest impact of noise on the spiking patterns and estimation performance.

Variation in firing period greatly influenced estimation performance (Figure 5D vs. Figure 5E, Figure 6D vs. Figure 6E, Figure 7C vs. Figure 7E, and Figure 7D vs. Figure 7F). Wide sampling of data on phase relations is required to improve Bayesian estimation. In the case of an identical firing period (σ_I_ = 0), the neurons exhibited in-phase synchronization (Figure 2A) or phase-locking with finite phase differences (Figure 3A). This limited sampling to specific phase differences. As a result, the estimation performance for the former case was the lowest among the examined conditions (Figures 5D, 7C,D). This is because when the neurons were globally synchronized, all phase differences were kept at 0. This led to biased sampling of phase relations, which is supported by the fact the confidence intervals did not converge, even for the larger cycles (Figures S1A,B). In contrast, when the variability of the firing period was not zero, but was small, it was possible to collect phase information for neuron pairs evenly over a cycle due to the slightly different firing periods. For the latter case, the phase differences for many neuron pairs were kept fixed, although those for some pairs slightly fluctuated due to random connections. This prevented us from sampling only limited phase differences. Thus, except in the special case of global synchrony, the proposed method performed well when sufficiently large and stationary spike data were available.

Partial observation is essential because it is impossible in principle to observe the activity of all neurons in the brain. Indeed, as the number of sampled neurons decreased, estimation performance deteriorated (Figures 7A,B). This is partially because the probability of sampling connected neuron pairs was decreased. If spikes of presynaptic neurons that contribute to the postsynaptic dynamics are missing (i.e., many Γ_*ij*_(Δϕ_*ij*_)s in Equation (2) are missing), the method would be forced to make them up, and consequently to make a wrong estimation. However, in the model investigated here, utilization of more data could serve as partial compensation. The present network model was a very small network compared to real neuronal networks. When applying the proposed method to a real neuronal network, the fraction of observable neurons should be very small. The analyses for the networks with different sizes also exhibited the same tendency that the estimation performance would deteriorate with a decrease in the fraction of observable neurons (Figures 7G,H) although we should note that the result was partly due to weakening of synaptic strengths. Thus, considering the analysis of spike data from a real neuronal network that consists of thousands or tens of thousands of neurons, the present method would be not practical and some improvements should be required. Possibly, as discussed above, the estimation performance might be improved if we can use the knowledge on anatomical connectivity to avoid randomly sample neurons to be recorded.

In the network model, neurons were connected by synapses of the same strength (synaptic conductance) or were unconnected, allowing the successful estimation of the interaction function and inference of synaptic connections so long as spike data from all neurons were used (Figure 8). Such binary connectivity potentially oversimplifies synaptic connectivity, given that synaptic strength is known to vary (Song et al., 2005). The magnitude of the interaction function is in proportion to the corresponding synaptic conductance. When using differing synaptic connection strengths, the interaction function of a weak synaptic connection could be estimated, although a large amount of spike data is required. In practice, however, it should be difficult to estimate such a weak interaction function based on limited spike data. Furthermore, it is significantly harder to make inferences when using differing synaptic connection strengths because of the inherent difficulty in separating the distributions of the summed powers. In particular, it would be quite difficult to discriminate a neuron pair with a very weak synapse from a neuron pair without synapses. Distinguishing between weak and unconnected synapses as accurately as possible would be more useful when introducing group lasso regularization to the Bayesian method.

## Author contributions

KS conducted the data analyses and wrote the paper. TA designed the study and edited the manuscript. KK designed the study, conducted the simulations, and wrote the paper.

### Conflict of interest statement

The authors declare that the research was conducted in the absence of any commercial or financial relationships that could be construed as a potential conflict of interest.

## Abbreviations

FN: false negative
FP: false positive
GABA: gamma-aminobutyric acid
GP: globus pallidus
MCC: Matthew's correlation coefficient
TN: true negative
TP: true positive.
