# Supplementary material for: Bayesian Estimation of Phase Dynamics Based on Partially Sampled Spikes Generated by Realistic Model Neurons
Source: Front Comput Neurosci. 2018 Jan 8;11:116. doi: 10.3389/fncom.2017.00116 (PMC5766690; doi:10.3389/fncom.2017.00116)
Supplement: Supplementary file 1 [file DataSheet1.docx]

**Fig. S1.** Estimated interaction function for the globally synchronized state. *σ*_I_*  *and *σ*_N_ were set to 0.0 and 0.4, respectively. **A**. The estimated interaction functions of a connected neuron pair for various cycle numbers of data (blue lines). derived from the detailed model is also shown for comparison (dashed lines). **B**. The estimated interaction functions of an unconnected neuron pair. **C.** The averaged L^2^ distances over connected and unconnected neuron pairs. The bars and error bars indicate means and standard deviations, respectively. **D.** The averaged powers of estimated interaction functions over connected and unconnected neuron pairs. The power of estimated interaction was obtained using *d_ij_*(**0**, **c***_i_*,*_j_*) in eq. (17), where **0** represents [0, 0, 0, 0, …, 0, 0] in eq. (10).

**Fig. S2.** Estimated interaction function for a case of increased synaptic strength. The proposed method was applied to the spike data for case A and to the doubled synaptic conductance (*g*_syn_ = 0.04 mS/cm^2^). **A**. The estimated interaction functions of a connected neuron pair for various cycle numbers of data (blue lines). derived from the detailed model is also shown for comparison (dashed lines). **B**. The estimated interaction functions of an unconnected neuron pair. **C**. Comparison of the odd part of  between the estimated and theoretically obtained functions. As in Fig. 5c, the filled circles indicate stable phase differences while the open circles show unstable phase differences. **D.** Dependence of L^2^-distances on the standard deviation of the applied currents. *σ*_N_ was set to 0.4.

**Fig. S3.** Estimated interaction function for a case of increased synaptic strength. This figure is analogous to Fig. S2, but uses data from case B instead of case A. **A**. The estimated interaction functions of a connected neuron pair for various cycle numbers of data (blue lines). derived from the detailed model is also shown for comparison (dashed lines). **B**. The estimated interaction functions of an unconnected neuron pair. **C**. Comparison of the odd part of  between the estimated and theoretically obtained functions. As in Fig. 6c, the filled circles indicate stable phase differences while the open circles show unstable phase differences. **D.** Dependence of L^2^-distances on the standard deviation of the applied currents. *σ*_N_ was set to 0.4.
