# Supplementary figures and images for: Bayesian Estimation of Phase Dynamics Based on Partially Sampled Spikes Generated by Realistic Model Neurons
Source: Front Comput Neurosci. 2018 Jan 8;11:116. doi: 10.3389/fncom.2017.00116 (PMC5766690; doi:10.3389/fncom.2017.00116)

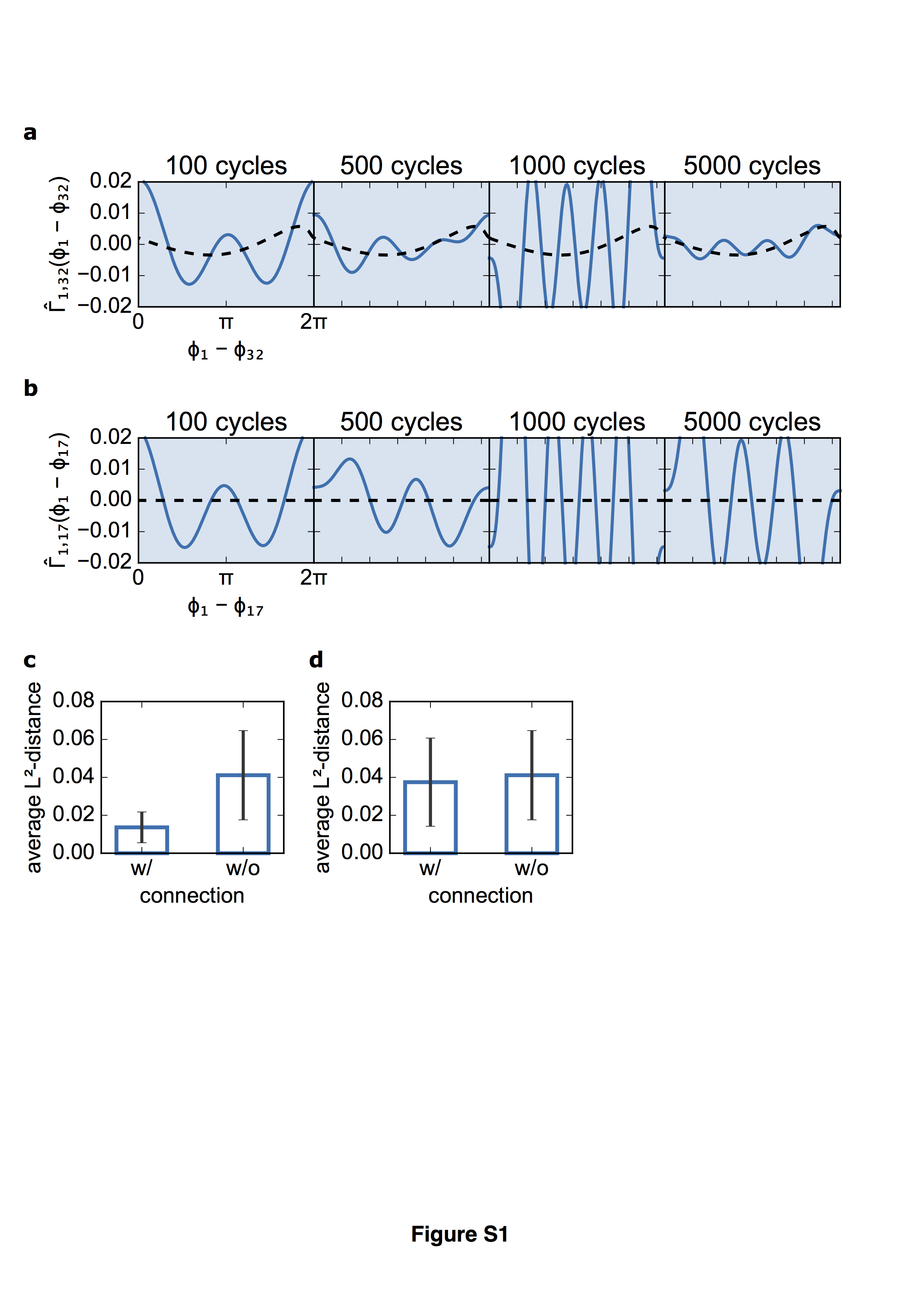

Supplement: Supplementary file 2 [file Image1.JPEG]

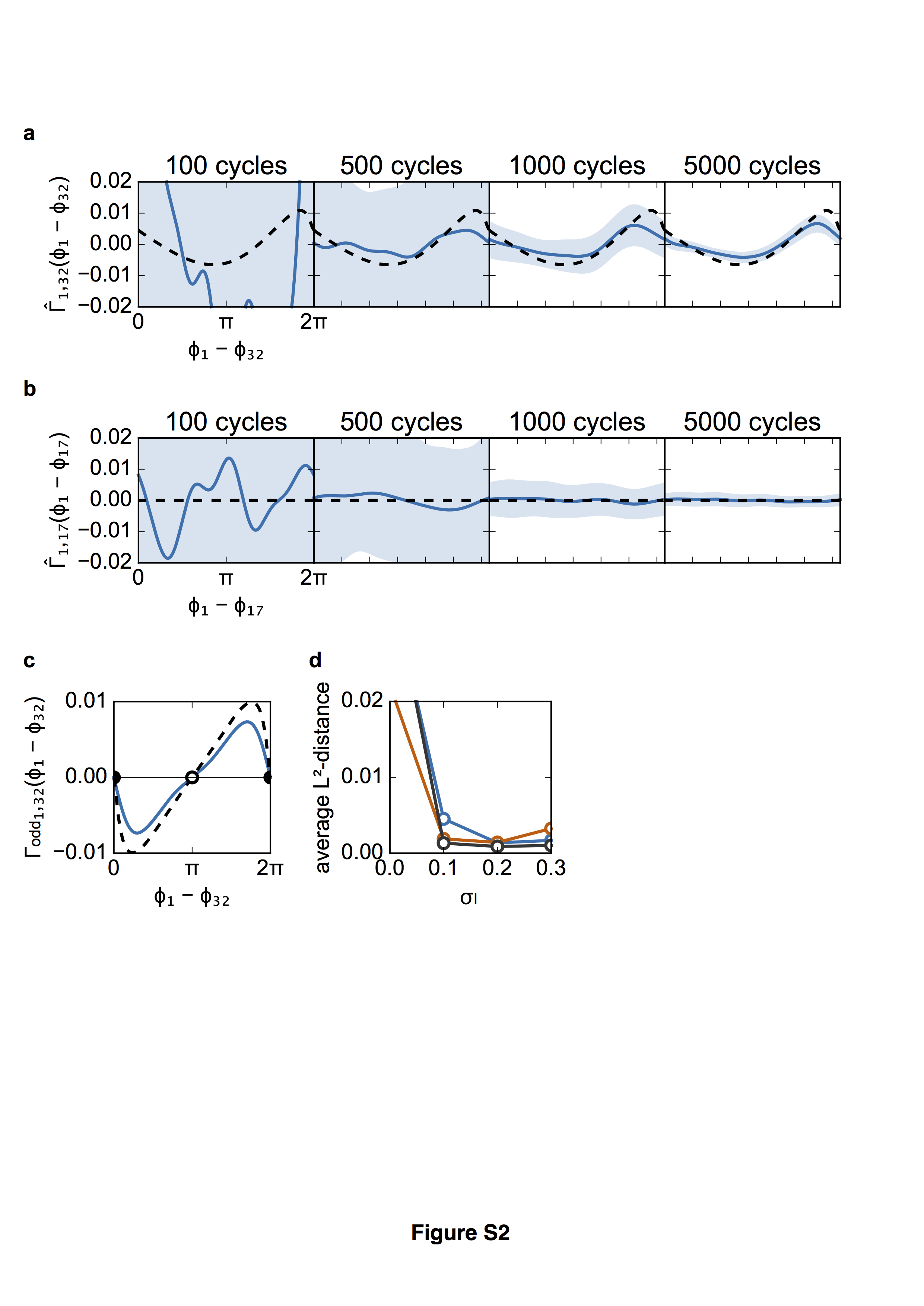

Supplement: Supplementary file 3 [file Image2.JPEG]

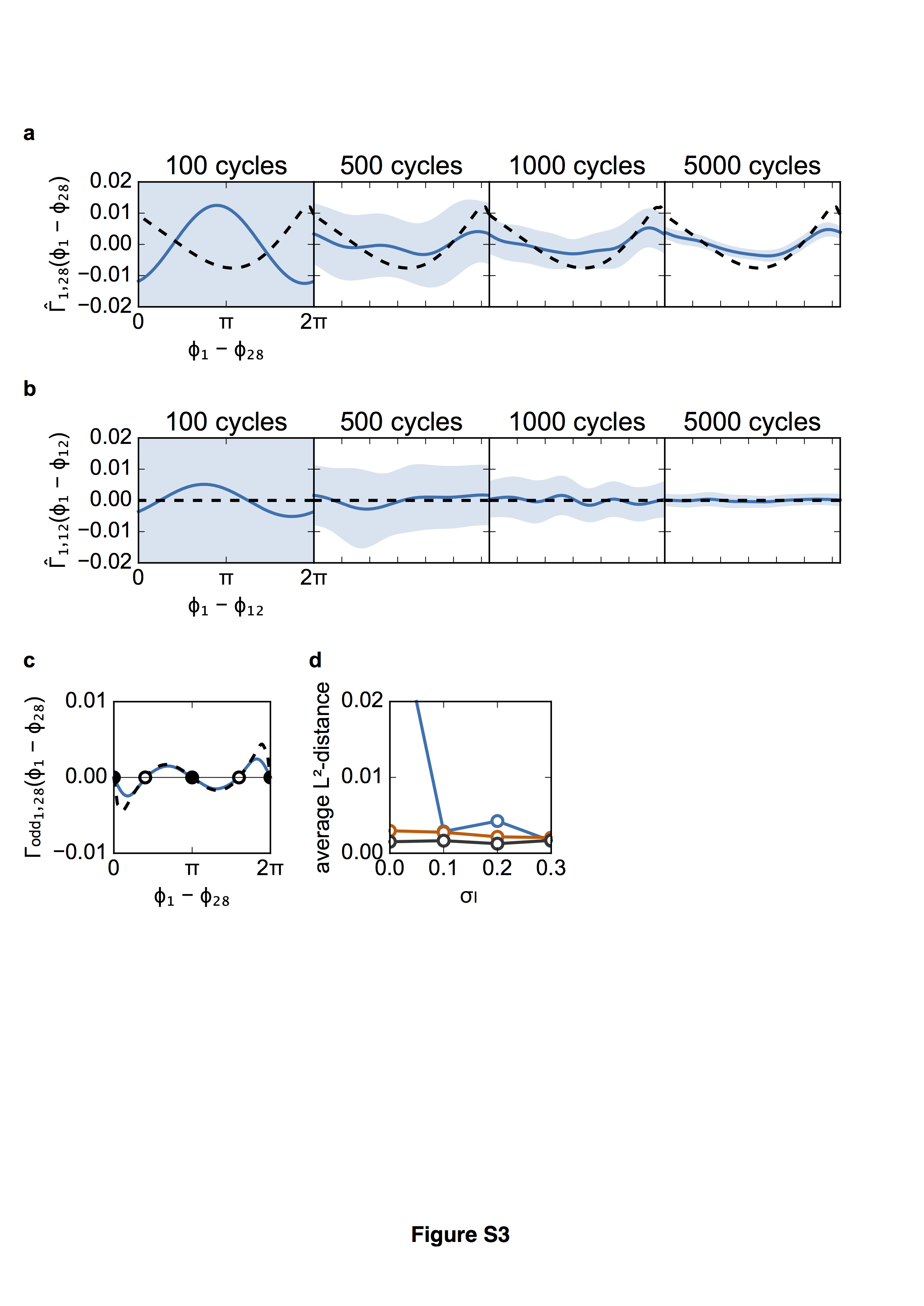

Supplement: Supplementary file 4 [file Image3.JPEG]
